# The Dynamics of Norovirus Outbreak Epidemics: Recent Insights

**DOI:** 10.3390/ijerph8041141

**Published:** 2011-04-15

**Authors:** John A. Marshall, Leesa D. Bruggink

**Affiliations:** Victorian Infectious Diseases Reference Laboratory, 10 Wreckyn Street, North Melbourne, Victoria 3051, Australia; E-Mail: leesa.bruggink@mh.org.au

**Keywords:** norovirus, outbreaks, epidemics, genetic factors, environmental factors

## Abstract

Noroviruses are a major cause of gastroenteritis outbreaks worldwide. Norovirus outbreaks frequently occur as epidemics which appear to be related to both genetic and environmental factors. This review considers recent progress in understanding these factors. The norovirus genome undergoes continuous change and this appears to be important in the persistence of the virus in the community. Studies on the common GII.4 genotype have shown that some norovirus outbreak epidemics involving this genotype are correlated with specific changes in the genome. In contrast to the growing understanding of the role of genetic factors in norovirus outbreak epidemics, the role of environmental factors is less well understood. Topics reviewed here include long term excretion of norovirus in some individuals, long term survivability of norovirus in the environment, the role of meteorological factors in the control of norovirus outbreaks and the possible zoonotic transmission of the virus.

## 1. Introduction

The noroviruses, which are single-stranded positive sense RNA viruses classified in the genus *Norovirus* within the family *Caliciviridae* [1], are now considered the most common cause of outbreaks of non-bacterial gastroenteritis worldwide [1], as well as being an important cause of sporadic gastroenteritis [1]. Epidemics of norovirus outbreaks, which can be defined as peaks in the incidence of outbreaks above the general level [2,3], are well established [3–5]. Norovirus outbreak epidemics can show an annual cyclic periodicity [3–5] and the dynamics of norovirus outbreak epidemics appear to be dependent on a complex interaction of different variables which include both genetic and environmental factors [4].

This review examines recent progress in understanding the roles of genetic and environmental factors in the control of the incidence of norovirus outbreak epidemics. The term “genetic” refers principally to nucleotide changes in the norovirus genome. Two main topics are reviewed here. Firstly, general aspects of genetic change in the norovirus genome and their relationship to altered infectivity/virulence of the virus are considered. Secondly, numerous recent studies linking genetic changes in the GII.4 genotype to changes in the pattern of outbreak epidemics linked to this genotype are reviewed. Topics reviewed in the category of “environmental factors” include norovirus excretion patterns, norovirus survivability in the environment, the role of meteorological factors in the control of norovirus outbreak epidemics and the possible zoonotic transmission of norovirus.

## 2. Genetic Factors

### 2.1. General

The norovirus genome is approximately 7.6 kb in length and typically comprises three open reading frames (ORFs) [1]. ORF 1 encodes the non-structural polyprotein, ORF 2 encodes the major structural capsid protein and ORF 3 encodes a small virion-associated protein [1]. Recent studies indicate that murine norovirus has an additional ORF, ORF 4, which overlaps ORF 2 [6].

Noroviruses are currently classified into five genogroups [7,8]. Three of these, genogroups I, II and IV (GI, GII and GIV), occur in human infections [9,10] though most noroviruses affecting humans belong to GI or GII [11]. Within each genogroup one or more “clusters” or “genotypes” have been identified [7,9,10]. In a major review of norovirus classification, Zheng *et al.* [7] examined 164 amino acid sequences from the capsid region of both human and animal noroviruses. These authors identified five genogroups comprising 29 genotypes, with eight genotypes in GI, 17 in GII, two in GIII, and one each in GIV and GV. More recently, Patel *et al.* [8] identified 32 genotypes among the five genogroups.

The major capsid protein encoded by ORF 2, which is referred to as VP1, is of considerable interest because it is believed to be involved in the recognition of the host receptor [12,13]. VP1 includes a shell and a protruding (P) domain, which, in turn, is made up of P1 and P2 subunits [10]. The P2 subunit is considered critical for receptor binding [10,12]. It has been proposed that amino acid changes in the P2 domain allow the virus to reinfect an individual (e.g., by host cell receptor switching) and thereby escape herd immunity [14].

The norovirus genome undergoes frequent change [15,16] by mechanisms including mutation [17] and recombination [18]. Although the relative importance of mutation *vs.* recombination in the evolution of norovirus remains unclear [19], there appears to be no doubt that recombination is important. For example, Bull *et al.* [18] identified 20 norovirus recombinant types in circulation worldwide and Motomura *et al.* [20] have suggested that recombination could be an important mechanism by which GII.4 remains persistent in human populations.

There is now some evidence that a small genetic change in the capsid region can have a big influence on norovirus virulence. Bailey *et al.* [21] found that murine norovirus virulence was linked to a glutamate to lysine substitution in the capsid region. Various nucleotide changes in other parts of the genome have also been linked to changes in norovirus virulence. For example, there is some evidence that nucleotide changes in the polymerase region of human GII.4 noroviruses may influence virulence [22–24].

### 2.2. Studies on GII.4 Variants

GII.4 norovirus is the most common cause of human norovirus outbreaks worldwide [25] and GII.4 noroviruses have been the focus of much genetic research. GII.4 strains frequently undergo genetic change and these altered forms are sometimes termed “variants” [25–27] or “subtypes” [20]. There is some evidence that the emergence of new GII.4 variants can correlate with the occurrence of norovirus outbreak epidemics. For example, the appearance of the GII.4 “Hunter” variant in Australia in 2004 coincided with an increase in norovirus outbreak activity [25]. Kroneman *et al.* [28] reported that an increase in gastroenteritis outbreaks in the Netherlands in 2004 was linked to the appearance of the GII.4 “GII.4-2004” variant. Ho *et al.* [29] reported a norovirus outbreak epidemic in 2006 in Hong Kong was linked to the emergence of the GII.4 “95/96-like” variant.

Variants have been identified as a cluster in either the GII.4 ORF 1 phylogenetic tree [3] or in the GII.4 ORF 2 phylogenetic tree [25]. In a study spanning 2002 to 2007 Bruggink and Marshall [3] found that each GII.4 norovirus outbreak epidemic was linked to one of four variants identified and there was a time link, a delay of 2 to 6 months, between the first detection of a GII.4 variant and the first outbreak epidemic in which it was the principal variant. It should be noted that the same GII.4 variant could be the principal variant in norovirus outbreak epidemics in successive years, so that the appearance of new variants cannot explain the annual cycle of GII.4 norovirus outbreak epidemics, thereby suggesting that environmental factors are important in the initiation of an epidemic (see Section 3.4).

## 3. Environmental Factors

### 3.1. General

Noroviruses can be spread by contaminated food or water, person-to-person contact [10] or aerosols [30,31]. Noroviruses are usually detected in faeces but have also been detected in vomitus [10] but apparently not in nasopharyngeal washings [10]. Norovirus-associated gastroenteritis outbreaks can occur in virtually any situation where groups of individuals gather together, including child-minding centres, school outings, camps, restaurants, hospitals, hostels, nursing homes, prisons and cruise ships [10,32,33]. The mode of spread of norovirus, its ubiquitous nature and the regular periodicity of norovirus outbreak epidemics [3–5] suggest environmental factors are important in the spread of the virus and some key topics here are considered next.

### 3.2. Norovirus Excretion Patterns

Norovirus excretion patterns may play an important role in the spread of the virus. Some key findings follow. There is now evidence that norovirus RNA or virion excretion can occur in the absence of major symptoms of gastroenteritis. For example, norovirus RNA or virion excretion can occur after major symptoms have abated [34–36] and there is a report in which norovirus RNA excretion was found in an individual the day before the onset of symptoms [36]. Furthermore, volunteer studies indicate that norovirus can be detected in infected individuals with no major symptoms [37] but studies of the asymptomatic excretion of norovirus RNA in naturally infected individuals indicate the percentage of asymptomatic excretors appears to depend on the population being sampled [38–40]. Norovirus excretion can continue for long periods. Norovirus has been detected up to seven days after inoculation of virus in one volunteer study [37] and norovirus RNA for up to 44.5 days following onset of symptoms in a naturally infected individual [41]. Prolonged norovirus RNA excretion is well documented in immunocompromised individuals [42–44].

### 3.3. Norovirus Survivability in the Environment

A number of studies have examined norovirus survivability in the environment. As human norovirus cannot be grown reliably in culture [45], a variety of approaches have been tried including the use of cultivable norovirus surrogates [46,47] or the application of a human norovirus “infectivity assay”, which does not involve a culture system [48]. Taken together all the approaches used indicate norovirus can survive for long periods in the environment. A brief review of some key studies follows.

Doultree *et al.* [46] used feline calicivirus as a norovirus surrogate in studies on the survivability of the virus in a variety of conditions. These authors found that the virus survived for at least 60 days at 4 °C with minimal loss of infectivity. At room temperature feline calicivirus was stable for 14 to 21 days in suspension in culture medium and 21 to 28 days in the dried state.

Bae and Schwab [47] compared the survivability of feline calicivirus with that of murine norovirus in water and concluded that murine norovirus had significantly higher rates of survivability than those of feline calicivirus. In particular, infectivity reduction at 25 °C was greater for feline calicivirus (0.18 log_10_/day) than for murine norovirus (0.09 log_10_/day). Similarly, nucleic acid reduction rates at 25 °C, determined using a multiple linear regression model, were higher for feline calicivirus (0.08 ± 0.03 log_10_/day) than for murine norovirus (0.04 ± 0.03 log_10_/day). However, caution is needed in assuming murine norovirus is the ideal surrogate for human norovirus in such studies. Tan and Jiang [13] and Lay *et al.* [49], for example, note that there appear to be important differences between human and murine noroviruses. These differences include the areas of clinical manifestation, host receptors, range of cell types where replication occurs and pathogenesis.

Lamhoujeb *et al.* [48] used human norovirus to study the survivability of norovirus on “food-contact” surfaces such as stainless steel and polyvinyl chloride. In their study, the identification of putatively infectious and noninfectious norovirus was based on an assay which utilized enzymatic pre-treatment of the virus such that noninfectious virus was believed to have been removed. On this basis the authors concluded that human norovirus could remain infectious for up to 28 days on both surface types at 20 °C.

### 3.4. Meteorological Factors

Currently there is little understanding of what environmental factors may correlate with the annual cycle of norovirus outbreak epidemics, but there is now some information on temperature and rainfall. Current evidence indicates temperature is not one of these factors, as norovirus outbreak epidemics tend to occur in colder months of the year in the northern hemisphere [50] and warmer months of the year in the southern hemisphere [4,32]. On the other hand there is evidence noroviruses can be spread by waterborne methods [51,52], thereby suggesting rainfall may be able to play a role in the control of norovirus outbreaks. Recent evidence supports this hypothesis. Bruggink and Marshall [53] found there was a statistically significant correlation between monthly norovirus outbreak incidence and average rainfall in a 2002–2007 study in Australia. It was found there was a lag of about three months between peak average rainfall and a norovirus outbreak epidemic following in the same calendar year. These results suggest that there is an environmental reservoir for norovirus and that rain, perhaps by altering the turbidity of reservoirs of water-borne norovirus, stimulates the spread of norovirus in the community and thereby helps initiate an outbreak epidemic.

### 3.5. Zoonotic Transmission of Norovirus?

Norovirus has been detected in a variety of animals including pigs [12,54,55], cattle [12], mice [12,56], dogs [57–60], sheep [55] and a lion [61]. The genogroups associated with human and animal noroviruses are as follows. GI norovirus has been linked to humans [57], GII norovirus to humans [57], pigs [57] and cattle [62], GIII norovirus to cattle [12,57,62] and sheep [55], GIV norovirus to humans [57], a lion [61] and dogs [57,60] and GV norovirus to mice [57]. It has been suggested that there is at least one additional norovirus genogroup, and that viruses in this group occur in dogs and humans [58,59].

A key public health question in this area is whether animals can act as a reservoir for human noroviruses. In a review of the literature, Koopmans [45] noted that, although zoonotic transmission of noroviruses had not been observed, the current understanding of norovirus epidemiology was too limited to be sure this did not occur.

## 4. Conclusions

It is now established that the norovirus genome undergoes frequent change and there is evidence that some of these changes are linked to altered viral infectivity. However, there is also evidence that environmental factors are important in the spread of norovirus. Future studies should not neglect the potential importance of environmental factors in developing a full understanding of the dynamics of norovirus outbreak epidemics.
